# Exercising alone versus with others and associations with subjective health status in older Japanese: The JAGES Cohort Study

**DOI:** 10.1038/srep39151

**Published:** 2016-12-15

**Authors:** Satoru Kanamori, Tomoko Takamiya, Shigeru Inoue, Yuko Kai, Ichiro Kawachi, Katsunori Kondo

**Affiliations:** 1 Department of Preventive Medicine and Public Health, Tokyo Medical University, Tokyo, Japan; 2 Human Resource Management Department, ITOCHU Techno-Solutions Corporation, Tokyo, Japan; 3 Physical Fitness Research Institute, Meiji Yasuda Life Foundation of Health and Welfare, Tokyo, Japan; 4 Department of Social and Behavioral Sciences, Harvard School of Public Health, Boston, Massachusetts, USA; 5 Center for Preventive Medical Sciences, Chiba University, Chiba, Japan; 6 Center for Well-being and Society, Nihon Fukushi University, Aichi, Japan; 7 Department of Gerontology and Evaluation Study, Center for Gerontology and Social Science, National Center for Geriatrics and Gerontology, Obu city, Aichi, Japan

## Abstract

Although exercising with others may have extra health benefits compared to exercising alone, few studies have examined the differences. We sought to examine whether the association of regular exercise to subjective health status differs according to whether people exercise alone and/or with others, adjusting for frequency of exercise. The study was based on the Japan Gerontological Evaluation Study (JAGES) Cohort Study data. Participants were 21,684 subjects aged 65 or older. Multivariable logistic regression models were used to examine the association. The adjusted odds ratios (ORs) for poor self-rated health were significantly lower for people who exercised compared to non-exercisers. In analyses restricted to regular exercisers the ORs for poor health were 0.69 (95% confidence intervals: 0.60–0.79) for individuals exercising alone more often than with others, 0.74 (0.64–0.84) for people who were equally likely to exercise alone as with others, 0.57 (0.43–0.75) for individuals exercising with others more frequently than alone, and 0.79 (0.64–0.97) for individuals only exercising with others compared to individuals only exercising alone. Although exercising alone and exercising with others both seem to have health benefits, increased frequency of exercise with others has important health benefits regardless of the total frequency of exercise.

Physical activity has been demonstrated to have various health benefits^1^,^2^. The benefits of physical activity apply regardless of the context, i.e. whether it occurs as part of work, leisure, transport, or housework^3^. However, it remains unclear whether exercise is more beneficial for those exercising with others, compared to exercising alone (e.g. on the basement treadmill).

This question has been previously discussed by distinguishing physical activity into exercising alone versus with others^4^. The mechanisms for health benefits from exercising with others may include not only physiological effects through physical activity, but also psychological and social factors. A systematic review focusing on the psychosocial benefits of exercising with others revealed that working out with others may enhance social connectedness, social support, and peer bonding^5^. These social relationships have been shown in turn to have potential health benefits^6^,^7^, and exercising with others may therefore have extra health benefits compared to exercising alone.

However, few studies have examined the differences in health associations between exercising alone and exercising with others. One study conducted on middle-aged Japanese adults showed that there was statistically no difference in the incidence of poor mental health five years later between non-exercisers and those exercising mostly alone, while the incidence was lower among those exercising mostly with exercising others, compared to non-exercisers^8^. However, the study did not directly compare exercising alone and with others, and the analyses did not adjust for differences in the frequency of exercise. One cohort study examining older Japanese adults showed a higher risk of incident functional disability (hazard ratio was 1.29 (95% confidence intervals: 1.02–1.64)) among those who did not participate in a sports organization compared to those who did, even though both groups reported regular exercise^9^. A cross-sectional study in Australian adults showed that sports club participants resulted in more positive benefits for various aspects of quality of life than gymnasium participants or walking participants^10^. These studies suggest the possibility that exercising with others has additional health effects over and above exercising alone. However, exercising alone and exercising with others were not directly compared. We therefore sought to address this gap using cross-sectional data from a cohort of older Japanese adults.

Self-rated health is one subjective indicator that reflects overall health status. Self-rated health is commonly used as a health outcome because of its established validity as a predictor of mortality, regardless of other medical, behavioral, or psychosocial factors^11^. Therefore, the aim of this study was to examine whether the association of subjective health status to exercise differs according to exercising alone and/or with others, adjusting for frequency of exercise. We hypothesized that there would be a lower prevalence of poor self-rated health among those performing exercising with others compared to those only exercising alone, even after adjusting for frequency of exercise. Although the existing guideline on physical activity mentions intensity and duration^3^, it does not mention whether exercise should be performed alone or with others. If exercising with others is shown to have greater health benefits than exercising alone, this would suggest the importance of including a social interaction perspective in health promotion using physical activity.

## Methods

### Study sample

We used cross-sectional data from the baseline wave of the Japan Gerontological Evaluation Study (JAGES), which is a population-based survey of community-dwelling seniors^12^. The JAGES sample includes only those who did not already have functional disabilities at the baseline survey. Those without functional disabilities were defined as those without eligibility for receiving long-term public care insurance benefits. The cohort was established in 2010 to examine prospectively the determinants of healthy aging in a sample of individuals aged 65 years and older. Subjects were selected by random sampling in each municipality, using the residential registry in each locality as the sampling frame. The present analysis was based on a sub-sample of the JAGES cohort study as a national sample of 137,736 people in 30 municipalities across Japan (response rate: 71.1%). Questionnaires were sent to 38,724 people and responses were received from 27,684 (response rate: 71.5%). We excluded 6,000 respondents who did not respond to the questions on age, sex, self-rated health, frequency of exercising alone and with others, or need of assistance in activities of daily living (ADL). The final study population consisted of 21,684 subjects. Subjects comprised 10,390 men (47.9%) and 11,294 women (52.1%), with a mean age of 73.5 ± 6.0 years.

### Measures

#### Subjective health status

Subjects were asked, “How is your current health status?” with possible responses: excellent, good, fair, and poor. Dichotomisation of multinominal self-rated health is frequently used in studies and has been validated^13^. Based on the previous study, subjects who responded with “fair,” or “poor,” were combined to form our outcome variable. The test-retest reliability of self-rated health was shown to be good in a variety of subgroups by age and sex^14^. In addition, the criterion-related validity of self-rated health was shown to predict mortality in a review^11^, and similar results were also observed in older Japanese adults, regardless of age, marital status, health behaviors, symptoms of depression, and chronic co-morbid conditions^15^.

#### Exercising alone and exercising with others

**Figure 1:**
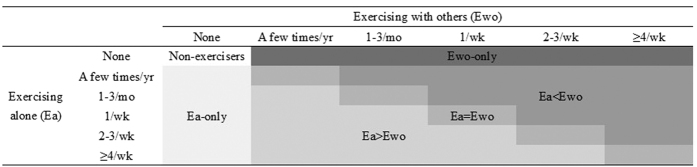
Patterns of exercise. Ea-only: people who only exercised alone. Ea > Ewo: people who reported exercising more frequently alone than with others. Ea = Ewo: people who reported exercising alone or with others with equal frequency. Ea < Ewo: people who exercised with others more frequently than exercising alone. Ewo-only: people who only exercised with others.

To define exercising alone, respondents were asked, “How often do you exercise alone?” To define exercising with others, respondents were asked, “How often do you exercise with a relative, friend, or acquaintance?” For each question, possible responses were: four or more times a week, two or three times a week, once a week, one to three times a month, a few times a year, and none. Based on a previous study that examined the relationship between mortality and physical activity^16^, the frequency of exercising alone and exercising with others was divided into six mutually exclusive categories: (1) non-exercisers, (2) people who only exercised alone (Ea-only), (3) people who reported exercising more frequently alone than with others (Ea > Ewo), (4) people who reported exercising alone or with others with equal frequency (Ea = Ewo), (5) people who exercised with others more frequently than exercising alone (Ea < Ewo); and (6) people who only exercised with others (Ewo-only) (Fig. 1). Next, the total frequency of exercise (combinations of two variable categories) was calculated and divided into six categories (see Supplementary Fig. S1). The higher the category, the greater the frequency of exercise. The categories were dichotomized into two groups: categories 1 to 3 reflected individuals who exercised less than twice a week, categories 4 and 5 exercised more than twice a week.

### Covariates

Based on previous studies^9^,^17^, age, sex, annual equivalized income (less than 2 million yen per year = “low”, 2–3.99 million yen per year = “middle”, 4 million yen or more per year = “high”), educational attainment (less than 10 years, more than 10 years), household composition (living alone, with others), occupational status (employed, not employed), self-reported medical conditions (no illness or disability, illness or disability), instrumental activities of daily living (IADL) (instrumental self-maintenance^18^; 5 points = “high”, 0–4 points = “low”), depressive symptoms (Geriatric Depression Scale^19^; 0–4 points = “no depression”, 5–9 points = “depressive tendency”, 10 points or more = “depression”), and total frequency of exercise were included as covariates in our regression models. Furthermore, as exercising with others may reflect sociability; frequency of meeting friends (two or more times a week, once a month to once a week, less than once a month), receiving instrumental support, providing instrumental support, receiving emotional support, and providing emotional support (yes, no) were also included as covariates.

### Statistical analysis

To examine whether the association of subjective health status to exercise differs according to exercising alone and/or with others, we performed multivariable logistic regression to calculate the odds ratios (ORs) for poor self-rated health. All variables were set as dummy variables. A “missing” category was used in analysis to account for missing values in response to questions.

The dependent variable was self-rated health and independent variables were the six groups characterized by frequency of exercising alone and exercising with others. In Model 1, age, sex, annual equivalized income, educational attainment, household composition, occupational status, self-reported medical conditions, IADL, depression, frequency of meeting friends, receiving instrumental support, providing instrumental support, receiving emotional support, and providing emotional support were added as covariates to the univariate model. In Model 2, total frequency of exercise was added to Model 1. In addition, to perform sensitivity analysis for examining whether the associations differ by total frequency of exercise, we conducted further analysis by stratifying the analyses into categories 4 and 5 (those who exercise at least twice a week) versus categories 1 to 3 (those who exercised less than twice a week).

SPSS 21.0 J was used for statistical analysis with a 2-tailed significance level set at 5%.

### Ethics statement

Ethical approval for the study was obtained from the Nihon Fukushi University Ethics Committee (application number: 10–04) and Chiba University Ethics Committee (application number: 1777). This study was performed in accordance with the principles of the Declaration of Helsinki. Informed consent was obtained from all participants.

## Results

Table 1 shows characteristics of individuals according to their patterns of exercise. Those who exercised with others (Ea > Ewo, Ea = Ewo, Ea < Ewo and Ewo-only) tended to be younger, and this group had a higher proportion of people with a high equivalized income, high educational attainment, living with others, high IADL score, no depression, rich social relationships, and good self-rated health. Among exercisers (Ea-only, Ea > Ewo, Ea = Ewo, Ea < Ewo and Ewo-only), there was a higher proportion of people who exercised less than twice a week among individuals who only exercised with others (Ewo-only).

Table 2 shows the adjusted ORs for poor self-rated health according to patterns of exercise. In Model 1 for all participants, the ORs for poor health were significantly lower for individuals who exercised (regardless of whether alone or with others; (Ea-only, Ea > Ewo, Ea = Ewo, Ea < Ewo and Ewo-only)). In the next set of models, we excluded non-exercisers in order to draw comparisons just among the different types of people who performed regular exercise. In these analyses, individuals who exercised alone (Ea-only) became the reference group for all comparisons. In Model 1, the ORs were 0.67 (95% confidence intervals: 0.58–0.77) for people who exercised alone more often than with others (Ea > Ewo), 0.72 (0.63–0.82) among people who exercised equally frequently alone or with others (Ea = Ewo), 0.58 (0.44–0.76) for individuals who exercised more often with others compared to alone (Ea < Ewo), and 0.86 (0.70–1.05) for individuals who only exercised with others (Ewo-only). The category of individuals who exclusively exercised with others (Ea > Ewo, Ea = Ewo, Ea < Ewo and Ewo-only) was statistically indistinguishable from people who exercised alone (Ea-only). The covariates in Model 1 plus total frequency of exercise were included in Model 2; the corresponding ORs were 0.69 (0.60–0.79), 0.74 (0.64–0.84), 0.57 (0.43–0.75), 0.79 (0.64–0.97).

Stratified analysis was then performed by dichotomizing the sample according to frequency of exercise. In Model 2, the ORs for individuals only exercising with others (Ewo-only) were similar results of the analysis performed on all exercisers, even though these were not statistically significant in either stratum.

## Discussion

This study was the first to examine whether the association of subjective health status to exercise differs according to exercising alone and/or with others, adjusting for frequency of exercise. As expected, in the analysis of all participants, the ORs for poor self-rated health were significantly lower for all exercise groups (Ea-only, Ea > Ewo, Ea = Ewo, Ea < Ewo and Ewo-only) compared to non-exercisers. In the analysis excluding non-exercisers, the ORs for poor self-rated health were significantly lower for people who exercised both alone and with others (Ea > Ewo, Ea = Ewo and Ea < Ewo) and people who only exercised with others (Ewo-only) compared to people who only exercised alone (Ea-only), after adjusting for total frequency of exercise. Moreover, although the ORs were not significantly lower for people who only exercised with others (Ewo-only), similar results were found when stratified analysis was performed using the collapsed groups reflecting frequency of exercise per week. These results imply that increased frequency of exercise with others has important health benefits regardless of the total frequency of exercise, although exercising alone and exercising with others both seem to have health benefits.

In a previous study on middle-aged adults, there was no difference between those who did not perform exercise or play sports (the reference category) and those who exercised mostly alone, whereas there was a significantly lower OR of poor mental health later on among those who exercised mostly with others^8^. Similarly, in a study on older adults, even for those exercising once a week or more, the risk of incident functional disability was significantly lower among those who participated in a sports organization compared to those who did not^9^. The results of these previous studies are consistent with the finding in the present study that the OR of poor self-rated health was significantly lower among those exercising with others than those only exercising alone.

In those who exercised with others, the ORs for poor self-rated health seem to be smaller for those exercising both alone and with others (Ea > Ewo, Ea = Ewo and Ea < Ewo) than those who only exercised with others (Ewo-only). This could still be residual confounding by total MET-hours, even though we were only crudely able to adjust frequency of exercise (i.e. those performing both may be likely to be spending more total time exercising compared to those only exercising alone). In contrast, the above-mentioned study on the association with mental health did not find any differences in the risk of poor mental health between those exercising both alone and with others and those who did not exercise *et al*.^8^. Although it is true that the reference for comparison was not the same, the trend observed was different from that of the present study. One reason for this difference may be that those exercising both alone and with others accounted for over half of those who exercised in the present study, which includes representative samples, but accounted for only 3% in the previous study.

Social relationships may be one mechanism underlying the health benefits of exercising with others^4^,^20^. Reviews have indicated that poor social relationships can increase mortality risk^6^,^7^, and similar results were also observed in older Japanese adults^21^. In addition, social connectedness while exercising contributes to exercise adherence^22^. Previous studies examining the mechanism underlying the relationship between exercising with others and health revealed the possibility that social relationships may contribute to the association between participation in a sports organization and incidence of functional disability^9^,^17^. In this research, we used a part of general social relationships (frequency of meeting friends, receiving instrumental support, providing instrumental support, receiving emotional support, and providing emotional support) as covariates which could serve as measures of sociability. As we could not use specific social relationships in exercising with others, future studies are needed to use specific social relationships in exercising with others to examine whether these social relationships mediate the association between exercising with others and health. Other possible mechanisms that may have a positive association with exercising with others are: adherence to exercise routines^23^,^24^,^25^, self-esteem and other psychological factors^5^, social capital^26^ and other social factors^4^. For example, those who exercise with others may have continued to exercise for more years at the time of the survey than those who exercise alone. As we could not determine the roles of those factors in the present study, further studies are needed.

The present study had some limitations. Firstly, while we considered the frequency of exercise, which is an important point when investigating the association between exercise and health, we did not consider intensity or duration^3^, or type of exercise^27^. The differences between exercising alone and exercising with others may be residually confounded by differences in these factors. The second limitation is that the phrase “exercise with others” did not differentiate between exercise with only one other person and exercise with two or more other people or in a group or organization. Associations with health may differ between the different forms of exercise with others. The third is that we used combinations of two variable categories for “total frequency of exercise”, which may have resulted in a slight lack of accuracy. The fourth is that there may be a confounding effect from demographic and psychosocial factors related to exercising with others^28^, which we did not examine. The fifth is that the study was cross-sectional, and therefore cannot determine causal relationships. Further studies are therefore also needed to consider these points.

## Conclusion

Among older Japanese adults, although exercising alone and exercising with others both seem to have health benefits, increased frequency of exercise with others has important health benefits regardless of the total frequency of exercise. A social interaction perspective may be useful to assist with promoting exercise benefits for older adults.

## Additional Information

**How to cite this article**: Kanamori, S. *et al*. Exercising alone versus with others and associations with subjective health status in older Japanese: The JAGES Cohort Study. *Sci. Rep.* **6**, 39151; doi: 10.1038/srep39151 (2016).

**Publisher's note:** Springer Nature remains neutral with regard to jurisdictional claims in published maps and institutional affiliations.

## Supplementary Material

Supplementary Fig. S1

## Figures and Tables

**Table 1:** Characteristics of individuals according to patterns of exercise.

		Ea-only	Ea > Ewo	Ea = Ewo	Ea < Ewo	Ewo-only	Non-exercisers
N	Mean ± SD	6,018	3,685	3,895	760	1,131	6,195
Age (years)		73.8 ± 6.1	72.6 ± 5.4	72.9 ± 5.4	72.3 ± 5.1	72.4 ± 5.5	74.5 ± 6.7
Sex (%)	Males	48.8	53.4	46.9	44.1	36.5	47.1
Equivalized income (%)	Low	44.3	38.2	41.7	41.1	35.2	44.1
	Middle	30.7	36.9	32.2	36.6	38.2	27.3
	High	7.9	10.6	9.2	10.3	10.8	8.5
	Missing	17.2	14.3	16.9	12.1	15.8	20.1
Educational attainment (%)	≤9	40.1	30.8	37.6	29.3	29.9	47.9
	≥10	58.5	68.1	61.2	69.5	68.3	50.1
	Missing	1.4	1.1	1.2	1.2	1.8	2.1
Household composition (%)	Living alone	16.8	12.0	11.1	11.2	11.4	13.6
	With others	79.1	84.8	85.0	85.7	85.5	81.3
	Missing	4.1	3.2	3.9	3.2	3.1	5.1
Occupational status (%)	Employed	22.2	20.8	22.2	17.4	22.9	26.1
	Not employed	70.1	72.5	70.1	76.1	70.5	63.6
	Missing	7.7	6.7	7.7	6.6	6.6	10.3
Self-reported medical condition (%)	No illness or disability	14.4	16.9	16.4	12.8	18.2	14.2
	Illness or disability	81.3	78.3	77.8	80.5	75.2	80.4
	Missing	4.3	4.8	5.8	6.7	6.6	5.4
IADL (%)	High	81.2	86.8	84.9	90.4	86.4	70.4
	Low	16.6	11.8	13.1	7.6	11.9	26.4
	Missing	2.2	1.4	2.1	2.0	1.7	3.2
Depression (%)	No depression	60.4	72.2	72.1	72.5	68.8	53.5
	Depressive tendency	18.5	11.3	12.2	12.1	13.4	20.9
	Depression	5.6	2.0	2.7	2.8	4.1	8.7
	Missing	15.4	14.4	13.0	12.6	13.8	16.9
Frequency of meeting friends (%)	<1/mo	31.9	16.1	16.3	13.8	16.9	35.6
	1/mo-1/wk	35.4	38.7	29.4	29.7	33.6	31.6
	≥2/wk	28.5	42.9	51.2	54.5	47.3	26.7
	Missing	4.2	2.3	3.1	2.0	2.2	6.2
Receiving emotional support (%)	Yes	91.3	95.7	95.7	97.1	95.8	89.3
	No	6.8	2.7	2.9	1.8	2.9	7.9
	Missing	1.9	1.6	1.4	1.1	1.3	2.8
Providing emotional support (%)	Yes	89.3	94.7	94.2	95.9	94.3	85.1
	No	7.6	3.0	3.5	2.1	3.7	10.6
	Missing	3.0	2.4	2.2	2.0	1.9	4.2
Receiving instrumental support (%)	Yes	91.7	95.6	96.1	97.0	95.5	91.8
	No	6.3	2.6	2.5	1.8	3.3	5.7
	Missing	2.0	1.8	1.4	1.2	1.2	2.4
Providing instrumental support (%)	Yes	75.3	82.5	81.5	82.2	83.3	72.1
	No	19.8	13.6	14.0	14.2	13.9	21.9
	Missing	4.9	3.9	4.5	3.6	2.8	6.0
Frequency of exercising alone (%)	None	0.0	0.0	0.0	0.0	100.0	100.0
	A few times/yr	7.8	0.0	13.4	24.3	0.0	0.0
	1–3/mo	8.1	4.9	9.7	26.7	0.0	0.0
	1/wk	11.4	9.4	12.3	29.6	0.0	0.0
	2–3/wk	27.3	31.4	29.8	19.3	0.0	0.0
	≥4wk	45.5	54.3	34.7	0.0	0.0	0.0
Frequency of exercising with others (%)	None	100.0	0.0	0.0	0.0	0.0	100.0
	A few times/yr	0.0	30.9	13.4	0.0	20.4	0.0
	1–3/mo	0.0	28.0	9.7	7.0	16.0	0.0
	1/wk	0.0	26.2	12.3	14.5	18.5	0.0
	2–3/wk	0.0	14.8	29.8	35.9	22.9	0.0
	≥4wk	0.0	0.0	34.7	42.6	22.2	0.0
Total frequency of exercise (%)	Non-exercisers	0.0	0.0	0.0	0.0	0.0	100.0
	Category 1	7.8	0.0	0.0	0.0	20.4	0.0
	Category 2	8.1	4.9	13.4	7.0	16.0	0.0
	Category 3	11.4	9.4	9.7	14.5	18.5	0.0
	Category 4	27.3	31.4	12.3	35.9	22.9	0.0
	Category 5	45.5	54.3	64.5	42.6	22.2	0.0
Self-rated health (%)	Poor	18.1	10.0	11.0	8.9	12.6	24.7

Ea-only: people who only exercised alone. Ea > Ewo: people who reported exercising more frequently alone than with others. Ea = Ewo: people who reported exercising alone or with others with equal frequency. Ea < Ewo: people who exercised with others more frequently than exercising alone. Ewo-only: people who only exercised with others. Total frequency of exercise (categories 1 to 3): people who exercised less than twice a week. Total frequency of exercise (categories 4 and 5): people who exercised at least twice a week. Results are presented as mean ± SD for continuous variables and percentage (%) for categorical variables.

**Table 2:** Odds ratios of poor self-rated health according to patterns of exercise.

	N	Crude		Model 1		Model 2	
		OR	95%CI	OR	95%CI	OR	95%CI
**All participants**							
Non-exercisers	6,195	ref	—	ref	—		
Ea-only	6,018	0.68	0.62–0.74	0.75	0.69–0.83		
Ea > Ewo	3,685	0.34	0.30–0.38	0.50	0.43–0.57		
Ea = Ewo	3,895	0.38	0.34–0.42	0.54	0.48–0.61		
Ea < Ewo	760	0.30	0.23–0.39	0.43	0.33–0.56		
Ewo-only	1,131	0.44	0.37–0.53	0.64	0.52–0.78		
**Exercisers-only: all participants excluding non-exercisers**							
**Total frequency of exercise: Category 1–5 (all exercisers)**							
Ea-only	6,018	ref	—	Ref	—	ref	—
Ea > Ewo	3,685	0.50	0.44–0.57	0.67	0.58–0.77	0.69	0.60–0.79
Ea = Ewo	3,895	0.56	0.50–0.63	0.72	0.63–0.82	0.74	0.64–0.84
Ea < Ewo	760	0.45	0.34–0.58	0.58	0.44–0.76	0.57	0.43–0.75
Ewo-only	1,131	0.66	0.54–0.79	0.86	0.70–1.05	0.79	0.64–0.97
**Total frequency of exercise: Category 4–5 (exercisers ≥2/wk)**							
Ea-only	4,381	ref	—	Ref	—	ref	—
Ea > Ewo	3,159	0.53	0.46–0.61	0.69	0.59–0.80	0.69	0.59–0.80
Ea = Ewo	2,995	0.57	0.50–0.65	0.73	0.62–0.85	0.76	0.65–0.90
Ea < Ewo	597	0.45	0.33–0.61	0.56	0.40–0.77	0.55	0.40–0.75
Ewo-only	510	0.60	0.45–0.80	0.80	0.58–1.09	0.78	0.57–1.06
**Total frequency of exercise: Category 1–3 (exercisers <2/wk)**							
Ea-only	1,637	ref	—	ref	—	ref	—
Ea > Ewo	526	0.54	0.41–0.71	0.72	0.54–0.97	0.69	0.51–0.93
Ea = Ewo	900	0.55	0.44–0.69	0.69	0.54–0.88	0.65	0.51–0.84
Ea < Ewo	163	0.46	0.28–0.76	0.65	0.39–1.10	0.62	0.37–1.05
Ewo-only	621	0.59	0.46–0.76	0.77	0.59–1.02	0.79	0.60–1.04

Ea-only: people who only exercised alone. Ea > Ewo: people who reported exercising more frequently alone than with others. Ea = Ewo: people who reported exercising alone or with others with equal frequency. Ea < Ewo: people who exercised with others more frequently than exercising alone. Ewo-only: people who only exercised with others. Model 1 was adjusted for sex, age, equivalized income, educational attainment, household composition, occupational status, self-reported medical conditions, IADL, depression, and sociability. Model 2 was adjusted for the covariates in Model 1 plus total frequency of exercise.
