# Supplementary material for: Exercising alone versus with others and associations with subjective health status in older Japanese: The JAGES Cohort Study
Source: Sci Rep. 2016 Dec 15;6:39151. doi: 10.1038/srep39151 (PMC5156899; doi:10.1038/srep39151)
Supplement: Supplementary Fig. S1 [file srep39151-s1.pdf]

Supplementary Information

Exercising alone versus with others and associations with subjective health status in older Japanese: The JAGES Cohort Study

Satoru Kanamori[1,2], Tomoko Takamiya[1*], Shigeru Inoue[1], Yuko Kai[3], Ichiro Kawachi[4], and Katsunori Kondo[5,6,7]

[1]Department of Preventive Medicine and Public Health, Tokyo Medical University, Tokyo, Japan

[2]Human Resource Management Department, ITOCHU Techno-Solutions Corporation, Tokyo, Japan

[3]Physical Fitness Research Institute, Meiji Yasuda Life Foundation of Health and Welfare, Tokyo, Japan

[4]Department of Social and Behavioral Sciences, Harvard School of Public Health, Boston, Massachusetts, United States of America

[5]Center for Preventive Medical Sciences, Chiba University, Chiba, Japan

[6]Center for Well-being and Society, Nihon Fukushi University, Aichi, Japan

[7]Department of Gerontology and Evaluation Study, Center for Gerontology and Social Science, National Center for Geriatrics and Gerontology, Obu city, Aichi, Japan

E-mail addresses

satoru_kanamori@hotmail.com (S. Kanamori)

takamiya@tokyo-med.ac.jp (T. Takamiya)

inoue@tokyo-med.ac.jp (S. Inoue)

y-kai@my-zaidan.or.jp (Y. Kai)

IKAWACHI@hsph.harvard.edu (I. Kawachi)

kkondo@chiba-u.jp (K. Kondo)

Corresponding author:

Tomoko Takamiya

Department of Preventive Medicine and Public Health, Tokyo Medical University

6-1-1 Shinjuku, Shinjuku-ku, Tokyo, 160- 8402, Japan

Phone: +81-3-3351-6141, Fax: +81-3-3353-0162

E-mail address: takamiya@tokyo-med.ac.jp

		Exercising with others (Ewo)					
		None	A few times/yr	1-3/mo	1/wk	2-3/wk	≥4/wk
	None	Non-exercisers	Category 1	Category 2	Category 3	Category 4	Category 5
Exercising alone (Ea)	A few times/yr						
	1-3/mo						
	1/wk						
	2-3/wk						
	≥4/wk						

Supplementary Fig. S1: Total frequency of exercise

Categories 1 to 3: people who exercised less than twice a week

Categories 4 and 5: people who exercise at least twice a week
